# Case Report: Inguinal Myxofibrosarcoma Arising From the Surgical Site of Resected Squamous Cell Carcinoma

**DOI:** 10.3389/fonc.2022.894421

**Published:** 2022-05-04

**Authors:** Hongyu Hu, Xianwen Hu, Dandan Li, Jiong Cai, Pan Wang

**Affiliations:** ^1^ Department of Nuclear Medicine, Affiliated Hospital of Zunyi Medical University, Zunyi, China; ^2^ Department of Obstetrics, Zunyi Hospital of Traditional Chinese Medicine, Zunyi, China

**Keywords:** myxofibrosarcoma, squamous cell carcinoma, magnetic resonance imaging, positron emission computed tomography, case report

## Abstract

Myxofibrosarcoma (MFS) is a rare soft tissue sarcoma that originates in the mesenchymal tissue and occurs mainly in the limbs of elderly patients. Herein, we present the case of a 64-year-old woman who underwent extensive left vulvar resection and bilateral lymphadenectomy for vulvar squamous cell carcinoma 6 months previously. A mass was found again at the original surgical site of the left groin 3 months prior, and its size had increased significantly in the past 1 month, with ulceration and pus. Magnetic resonance imaging (MRI) showed a 10-cm mass in the left groin area; fluoro18-labeled deoxyglucose positron emission tomography/computed tomography (^18^F-FDG PET/CT) showed a marked increase in mass metabolism in the left groin area, which was highly suspected to be a recurrence of squamous cell carcinoma. Subsequently, the patient underwent surgery and the postoperative pathology and immunohistochemistry confirmed MFS. In conclusion, MFS has rarely been reported to arise from the surgical site of squamous cell carcinoma. Our case study demonstrates that MFS should be included in the differential diagnosis of superficial masses in patients with a prior surgical history who present with a soft tissue mass at the surgical site, especially for recently developed rapidly increasing masses. This study aimed to systematically review the clinical features, diagnosis, differential diagnosis, treatment, and prognosis of this disease based on our case and related published literature and to provide clinicians with a broader perspective on the differential diagnosis of soft tissue tumors.

## Case Description

**Figure 1:**
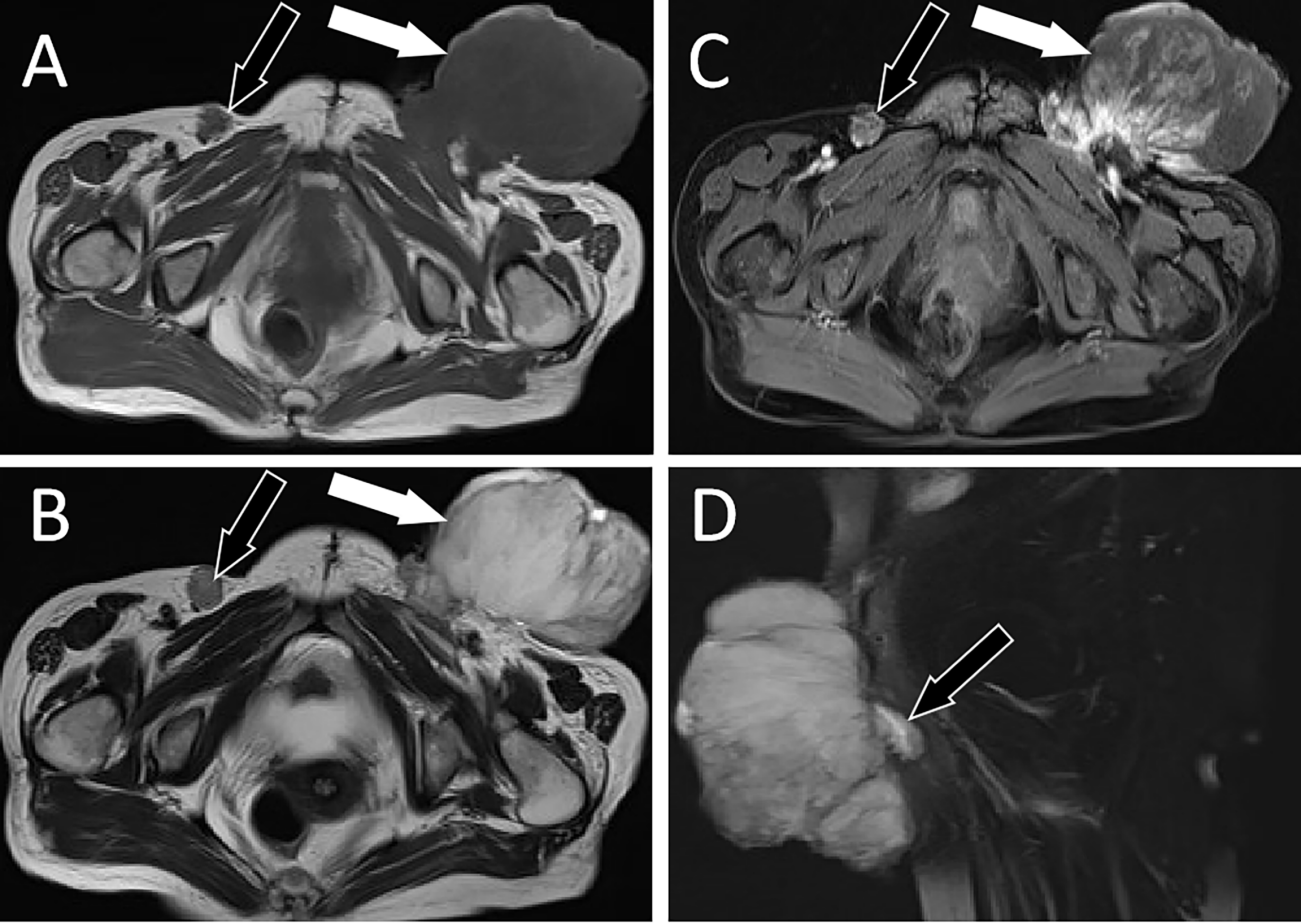
MRI examination; **(A)** Axial T1WI shows hypointense signal of left groin mass (white arrow) and right inguinal lymph nodes (black arrow). **(B)** Axial T2WI imaging demonstrates slightly high signal intensity in the left groin area (white arrow) and low signal intensity in the right inguinal lymph nodes (black arrow). **(C)** Contrast-enhanced T1WI with fat suppression: the left groin mass showed uneven enhancement (white arrow), while the right inguinal lymph nodes showed marginal enhancement (black arrow). **(D)** Sagittal contrast-enhanced T1WI clearly shows the “tail fascial sign” (black arrow). T1WI, T1-weighted images; T2WI, T2-weighted images.

**Figure 2:**
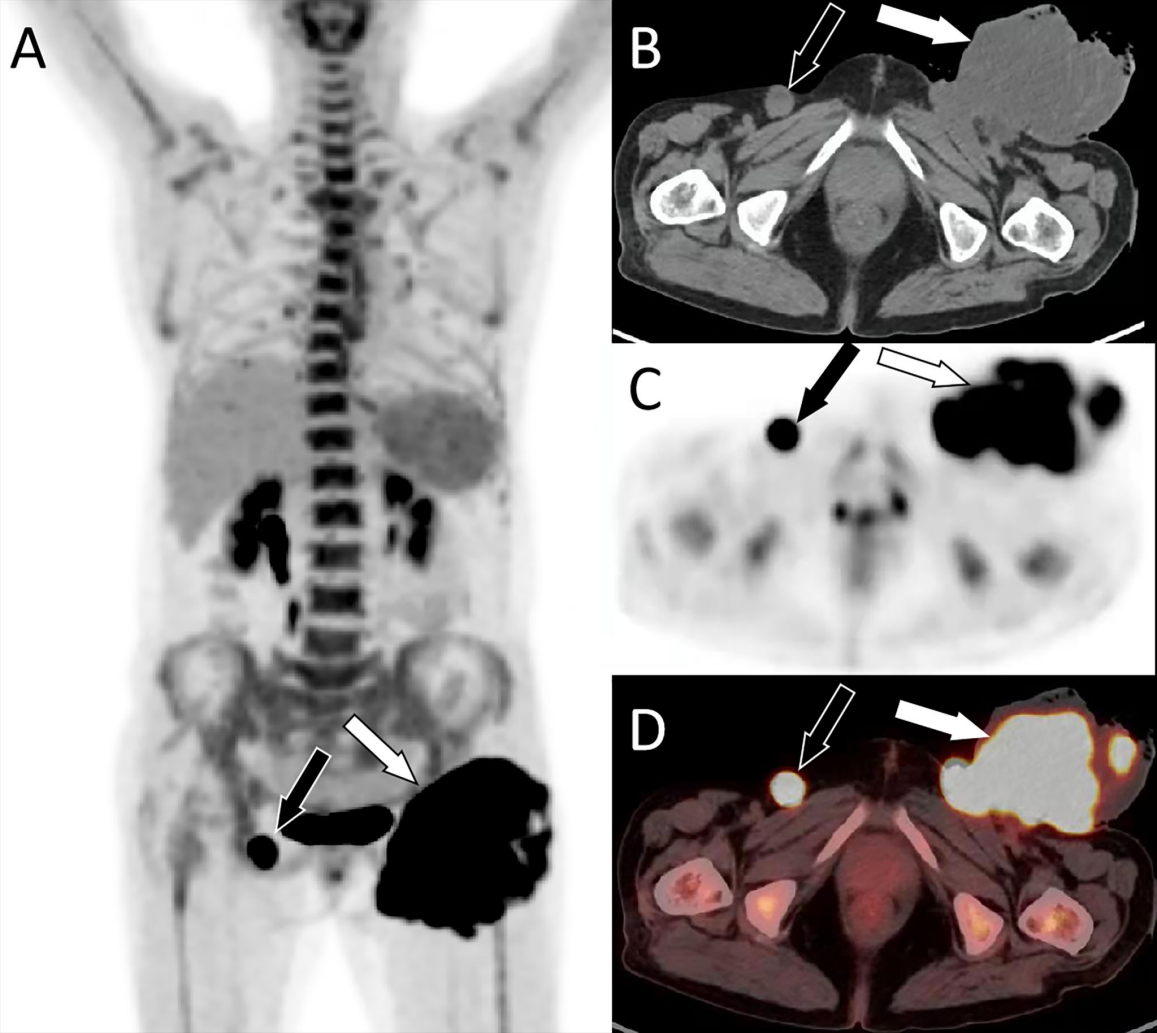
PET/CT examination. **(A)** Whole-body MIP images showed a lumpy hypermetabolic lesion in the left groin (white arrow) and increased nodular uptake of FDG in the right groin (black arrow). **(B)** Axial CT shows an uneven low-density mass in the left groin (white arrow) and a rounded soft tissue density nodule in the right inguinal region (black arrow). **(C)** PET and **(D)** PET/CT fusion images show a hypermetabolic mass in the left groin area with SUVmax of 31.38 (white arrow) and nodules in the right groin area with radioactive uptake with SUVmax of 28.0 (black arrow). MIP, maximum intensity projection; FDG, fluorodeoxyglucose; PET, positron emission tomography; CT, computed tomography; SUVmax, maximum standard uptake.

**Figure 3:**
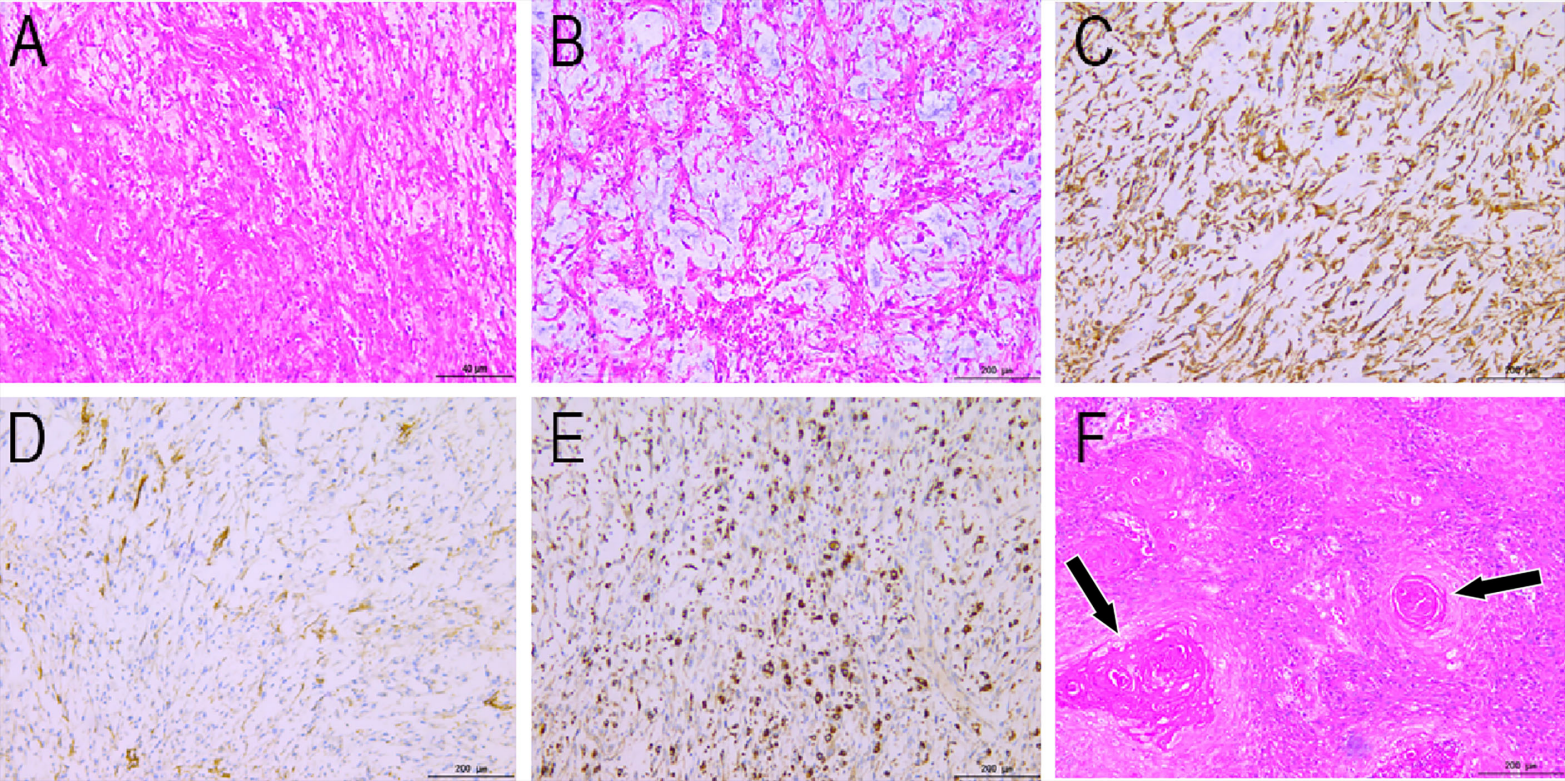
Histopathological findings (HE staining: **A**, ×40; **B**, ×200) show fusiform tumor cells, curving vessels, and myxoid alterations in the stroma. Immunohistochemical staining showing vimentin **(C)**, SMA **(D)**, and CD68 **(E)** positivity. **(F)** Histopathological view of the right inguinal lymphadenopathy. HE staining shows irregular squamous cells and horn strains (arrows). HE, hematoxylin and eosin.

A 64-year-old woman was admitted to a local county hospital 6 months previously because of the discovery of a left vulvar mass. CT revealed a mass of approximately 3.5 cm × 2.0 cm (vertical diameter × horizontal diameter) in her left vulva, and multiple enlarged lymph nodes were seen in the left groin area (**Supplementary Figure S1**). The clinician suspected that she had a malignant tumor and lymph node metastasis in the left inguinal region; therefore, extensive excision of the left vulvar mass and repair of the vulva followed by bilateral lymph node dissection were undertaken. Postoperative pathology revealed keratinized squamous cell carcinoma of the vulva, with a tumor invasion depth of 5 mm, no tumor invasion at the resection margin, metastasis in the left inguinal lymph node (3/7), and no metastasis in the right inguinal lymph node (0/3). The patient did not undergo further postoperative chemoradiotherapy. She complained that a painful lump had reappeared in the original surgical incision area of her left groin area 3 months previously; in the past month, the lump had rapidly increased in size, ruptured, and ulcerated; thus, she visited our hospital for treatment. Physical examination revealed a cauliflower-like protruding skin mass in the left groin with superficial ulceration and bleeding. Moreover, a 2-cm mass was observed in the right inguinal area, and the skin surface was red without ulceration or exudation. She had no other positive signs, and her routine blood and tumor marker values were within the normal range. She then underwent imaging examinations; computed tomography (CT) demonstrated a soft tissue-dense mass in the left groin area, which presented as low T1 and high T2 signals on magnetic resonance imaging (MRI), and contrast-enhanced scan showed tumor infiltration into the fascia as “fascial tail sign.” In addition, an unevenly enhanced nodular abnormal signal shadow was observed in the right inguinal region, as shown in **Figure 1**. Fluoro18-labeled deoxyglucose positron emission tomography/computed tomography (^18^F-FDG PET/CT) showed a high FDG concentration in the left groin mass, with a maximum standard uptake value (SUVmax) of 31.38, and increased FDG uptake in the right nodule, with an SUVmax of 28, as shown in **Figure 2**. According to the patient’s history of vulvar squamous cell carcinoma and the above imaging findings, the radiologist first considered that the left mass was a recurrence of squamous cell carcinoma, while the right inguinal nodule was a lymph node metastasis. Therefore, the patient underwent enlarged resection of the tumor in the left inguinal region with local pedicled flap transfer and repair and bilateral inguinal lymph node dissection. During the operation, the scalpel moved the skin and subcutaneous tissue along a 2-cm incision at the edge of the tumor to the tumor base, and the tumor base was found to be adherent to the femoral artery and femoral vein. The transverse diameter of the tumor after resection was approximately 20.0 cm, and no tumor involvement was observed at the upper, lower, left, or right margins of the tumor under a microscope. Hematoxylin–eosin staining showed that the tumor cells in the left mass were spindle-shaped, the blood vessels were curvilinear, the interstitium was myxoid, and the right groin lesion showed an irregular squamous cell composition and visible horn strains. Immunohistochemistry showed positive expression of vimentin, CD68, and partial smooth muscle actin (SMA) and negative expression of β-catenin, CD, Cluster of differentiation (CD34), desmin, S100, and Signal transducer and activator of transcription (STAT6) in left groin tumor cells, as shown in **Figure 3**. Based on these pathological and immunohistochemical findings, the patient was diagnosed with myxofibrosarcoma (grade II) in the left groin region and keratinized squamous cell carcinoma in the right inguinal lymph node. The patient refused chemotherapy and radiotherapy after surgery; thus, we suggested a follow-up review. The patient was still alive after a 1-year follow-up, and the latest MRI results showed no tumor recurrence or local metastasis.

## Discussion

Myxofibrosarcoma is a mesenchymal tumor that most commonly occurs in the extremities of elderly patients aged 60–70 years, especially in the lower extremities. It rarely affects the trunk, head, neck, hands, or feet (1). Myxofibrosarcoma in the groin has rarely been reported in the literature. Clinically, compared with other types of sarcomas, MFS has a higher local recurrence rate and lower distant metastasis rate (2). Pathological examination usually classifies tumors into four grades of malignancy based on their cell abundance, cytopleomorphism, and prevalence of mitotic images (3). MFS has obvious atypia, and grade I and II tumors are dominated by fibroblast-like cells, while grade III and IV tumors are mainly characterized by histiocytic cells (4). Histologically, MFS is characterized by alternating hypocellular and myxoid areas, multicellular fibrous areas, long curvy blood vessels in various mucinous stroma, and a polynodular growth pattern (5). Tumors can be either shallow or deep and usually occur under or in the context of the skin, accounting for 20%–70% of the cases (6). The etiology of the disease is not clear at present, but the association between acute trauma and the development of soft tissue sarcoma was first reported in the literature in 1901, as well as in surgical scars, burn scars, and sites of repeated trauma (7). Ineffective wound repair by dysfunctional fibroblasts is thought to play a key role in accelerating the formation of malignant tumors in genetically predisposed individuals (8). It has also been reported that acute tissue injury activates satellite cells and promotes sarcoma formation through the HGF, Hepatocyte growtll factor (HGF)/C-MET signaling pathway (9). Clinically, cases of myxofibrosarcoma secondary to soft tissue injury have been reported (10–12). In the present case, the patient was tumor-susceptible, and the tumor occurred at the surgical site. Therefore, it was speculated that the occurrence of the tumor might be related to previous surgical trauma; however, a larger number of cases is necessary to confirm this hypothesis. Tumors are abundant in the myxoid matrix. Weiss and Enzinger, in their initial description of Malignant Fibrous Histiocytoma (MFH) myxoid variants, required that at least 50% of tumors should consist of myxoid regions in order for it to be classified as MFS (13). As the imaging presentation of this disease is not specific, it is difficult to make a specific diagnosis. Therefore, we lack a typical understanding of this disease, increasing the chances for misdiagnosis. However, imaging examination also has important clinical significance for fibrosarcoma, and common imaging examinations include CT, MRI, and PET-CT. MRI has a good resolution that can not only clearly show the location, size, shape, and invasion range of the tumor but also reveal the pathological components of the tumor; thus, it is considered to be an indispensable examination method for the diagnosis of soft tissue tumors, including myxofibrosarcoma (1). Depending on the components of the lesion, there are different manifestations on imaging. Due to the presence of the myxoid matrix in tumors, myxoid changes are relativistic on MRI. On T1-weighted imaging (T1WI), myxoid substrates with low cell density have less signal intensity than muscle, but areas with high cell density are similar to the intensity of muscle on MRI. The myxoma matrix has signal intensity on T2WI/Short Time Inversion Recovery (STIR), whereas areas with high cell density show low signal intensity, and myxoid matrix enhancement is poor (14). MFS is classified into two types, solid and “tail-like”, based on T2-weighted MRI. In the “tail-like” type, there is extensive spread along the fascial planes that extended away from the primary site of tumor (15). This type of tumor often infiltrates and extends along the fascia plane, showing a specific “tail fascia sign” on MRI, which is consistent with myxoid fibrosarcoma; however, not all cases of this histotype show this feature (14). PET/CT also has high value in revealing details on tumor metabolism and distant metastasis and has high sensitivity for the detection of primary sites and metastases. PET/CT is a new imaging method that plays a significant role in the detection, staging, and treatment of many sarcomas and cancers. However, the small sample size has not proven its effectiveness (16). The radioactive uptake of myxoid tumors is linked to the proportion of mucous components in the tumors. Generally, tumors with a higher proportion of mucus have a lower radioactive uptake, which is related to the fact that the mucous components of tumors cannot capture FDG (17). There are few reports on the ^18^F-FDG PET/CT findings of myxosarcoma, with a maximum standard value range of 10.1 to 16.8 (18–20). In this case, myxofibrosarcoma showed hypermetabolism on PET-CT, which may be related to the fact that our patient had grade II myxofibrosarcoma with more spindle cells and less myxoid matrix (approximately 60%). The clinical, pathological, and imaging features of the disease overlap with different histotypes, and an accurate diagnosis can be challenging. It is often necessary to distinguish it from other mucinous tumors such as intramuscular myxoma and myxoid liposarcoma. Intramuscular myxoma is a common benign myxoid soft-tissue tumor. On MRI, there is a feathery T2 hyperintensity around the lesion, often in a mildly diffuse or thick peripheral and septal pattern (21). Myxoid liposarcoma usually appears in younger patients and is characterized by its lipid content (22). Extraskeletal myxoid chondrosarcomas are distinguished by their characteristic cartilage matrix on MRI (23). The diagnosis of this disease relies mainly on histopathological examination, which is the gold standard. Surgical resection is the standard treatment for local disease. Generally, when surgery is performed, extensive resection should include a soft tissue edge of 2 cm around the tumor and tumor cells should not be left at the edge (24). Postoperative radiotherapy is essential when an adequate margin cannot be obtained (25). Patients with this disease have an overall 5-year survival rate of approximately 60%–70%, and good disease-specific survival compared to that seen in other sarcomas (26). Due to the high recurrence rate of this tumor, all patients require close observation and follow-up after treatment. Our patient was still alive after a 1-year follow-up by imaging examination, and the latest MRI results showed no tumor recurrence or local metastasis (**Supplementary Figure S2**).

In conclusion, myxofibrosarcoma rarely develops in the surgical region after squamous cell carcinoma. The presence of “tail fascial sign” on MRI suggests the possibility of the disease, and the radioactive uptake of tumors on PET-CT images is related to the composition of mucous in tumors. More mucus in tumors leads to low metabolism, while less mucus leads to high metabolism. Second, our case suggests that myxofibrosarcoma may be associated with surgical trauma; however, this needs to be confirmed in a large number of cases in the future. In addition, our patient had an incidental association of left groin myofibrosarcoma and right inguinal lymph node squamous cell carcinoma metastasis, suggesting that non-monism should be considered in the diagnosis of tumors in future studies.

## Data Availability Statement

The data involved in the article can be obtained through the corresponding author under reasonable conditions.

## Ethics Statement

The present study was approved by the Ethics Committee of Affiliated Hospital of Zunyi Medical University, Zunyi, Guizhou, China.

## Author Contributions

JC and PW: funding acquisition; DL: investigation; XH: methodology; HH: writing -original draft; HH and JC: writing—review and editing.

## Funding

This study was funded by the National Natural Science Foundation of the People’s Republic of China, NSFC (grant numbers: 81571712), Zunyi Medical College Research Start Fund 2018ZYFY03, and QianKeHe platform talents [2017] (grant numbers:5733-035).

## Conflict of Interest

The authors declare that the research was conducted in the absence of any commercial or financial relationships that could be construed as a potential conflict of interest.

## Publisher’s Note

All claims expressed in this article are solely those of the authors and do not necessarily represent those of their affiliated organizations, or those of the publisher, the editors and the reviewers. Any product that may be evaluated in this article, or claim that may be made by its manufacturer, is not guaranteed or endorsed by the publisher.
