# Supplementary figures and images for: Case Report: Inguinal Myxofibrosarcoma Arising From the Surgical Site of Resected Squamous Cell Carcinoma
Source: Front Oncol. 2022 May 4;12:894421. doi: 10.3389/fonc.2022.894421 (PMC9114730; doi:10.3389/fonc.2022.894421)

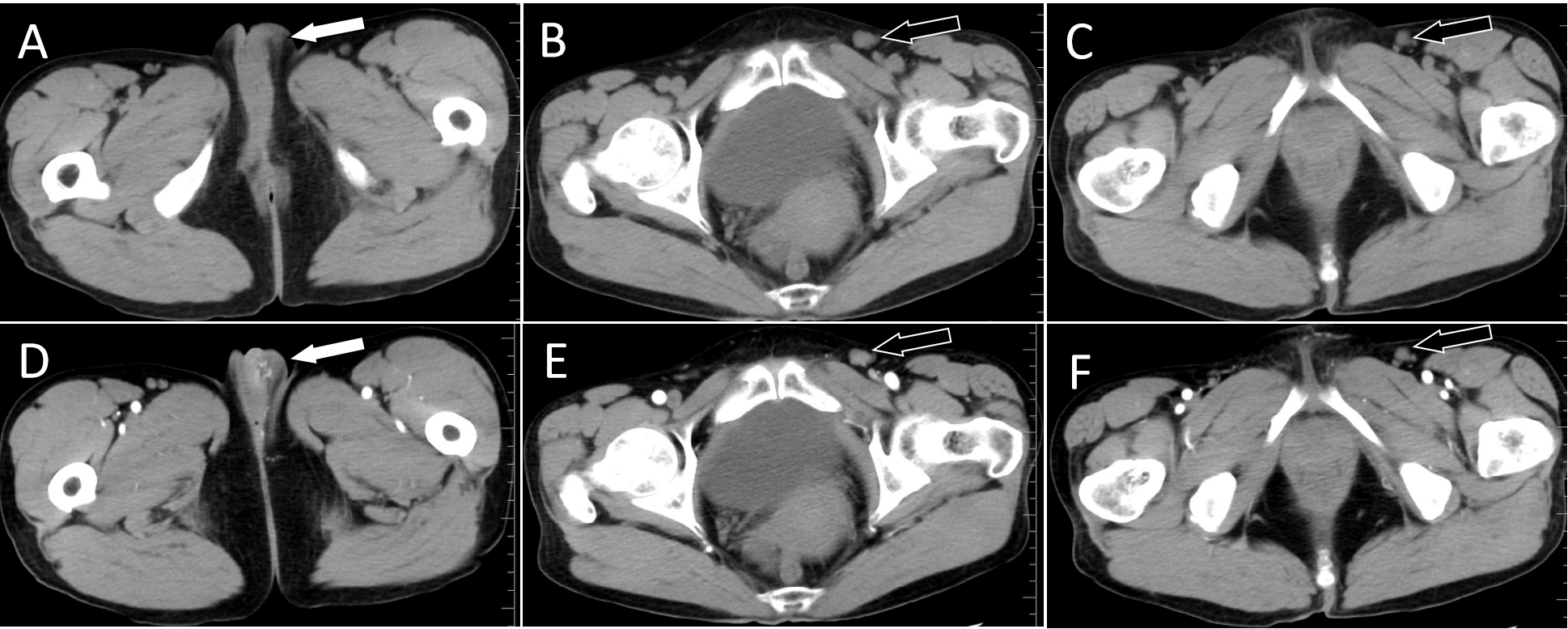

Supplement: Supplementary file 1 [file Image_1.tif]

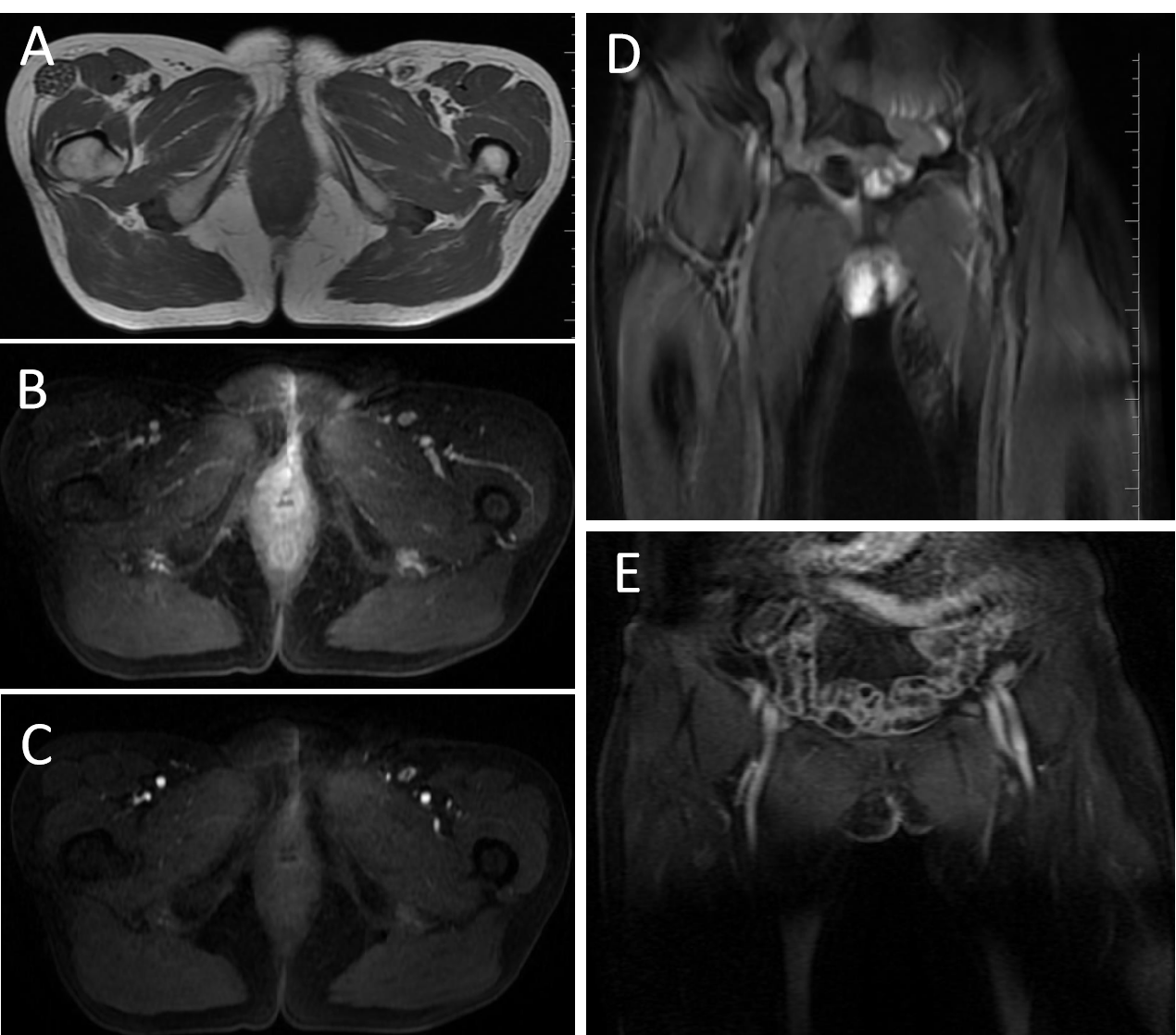

Supplement: Supplementary file 2 [file Image_2.tif]
